# Recent Advances in Sensor Technology for Healthcare and Biomedical Applications (Volume II)

**DOI:** 10.3390/s23135949

**Published:** 2023-06-27

**Authors:** Wenfeng Zheng, Mingzhe Liu, Chao Liu, Dan Wang, Kenan Li

**Affiliations:** 1 School of Automation, University of Electronic Science and Technology of China, Chengdu 610054, China; 2 College of Computer Science and Cyber Security, Chengdu University of Technology, Chengdu 610059, China; 3 French National Center for Scientific Research (CNRS), LIRMM, 34095 Montpellier, France; 4 Department of Internal Medicine, The Ohio State University, Columbus, OH 43210, USA; 5 Department of Epidemiology and Biostatistics, Saint Louis University, St. Louis, MO 63103, USA

With remarkable progress being witnessed in recent years in the development of sensors, these advances in sensor technology provide unprecedented opportunities for (1) the early diagnosis and prevention of human diseases by detecting critical biomarkers; (2) health assessments by monitoring and analyzing human physiological signals in healthcare and biomedical applications; and (3) the efficient evaluation of human-health-relevant environmental factors by monitoring and measuring environmental determinants.

This Special Issue aims to provide an overview of recent advances being made in sensing technology, including sensors and smart devices, and their applications in healthcare, biomedical, and environmental research. The purpose of this Special Issue is to publish a collection of papers that highlight insightful and influential concepts, designs, algorithms, and techniques in this field. We expect these high-quality papers to be widely read and cited for improving the understanding of physiological mechanisms through accurate and sensitive detection, providing new perspectives and technology to solve health-related problems and state-of-the-art designs to integrate multifunctional sensors.

Mingyu Sun et al. [1] proposed a scalable joint blind source separation (JBSS) method by modeling and separating the “shared” subspace from the data. Their method firstly involved the efficient initialization of the independent vector analysis (IVA) with prior use of a multivariate Gaussian source (IVA-G) specifically designed to estimate the shared sources. Estimated sources were then evaluated depending on whether they were shared, after which further JBSS was applied separately to the shared and non-shared sources. Independent vector analysis by shared subspace separation (IVA-S3) can achieve excellent estimation performances with significantly reduced computational costs.

Ramona B. Damalerio et al. [2] fabricated flexible dry electroencephalogram (EEG) electrodes with impedance values of <150 kΩ. Partially embedding the polyimide flexible printed circuit board in polydimethylsiloxane and Ag–AgCl coating at the tip of the electrode legs enabled steady low-contact-impedance readings. Universal single-piece and stretchable dry EEG head caps were also developed. The use of Velcro-compatible neoprene fabric facilitates the easy repositioning of the electrodes while still following the 10–20 standard placement. The findings of this study are useful, especially in areas where the extended recording of EEG measures is required. The developed dry electrode can be paired with various types of head caps and dry EEG systems for clinical settings, non-laboratory settings, and even in gaming.

Hamed Samimi and Hilmi R. Dajani [3] focused on a cuffless blood pressure estimation based solely on information carried by photoplethysmogram signals. This proposed alternative method used dynamic changes in the pulse waveform over short intervals, and replaced calibration with information from photoplethysmography (PPG) morphology to provide a calibration-free approach using a single sensor. They demonstrated that the cardiovascular dynamics contain useful information that can help to improve the accuracy of blood pressure estimation when features based on pulse morphology are also considered.

Haoran Liu et al. [4] proposed an image-fusion scheme based on the non-subsampled contourlet transform and spiking cortical model (NSCT-SCM) to combine the tri-contrast images retrieved from X-ray grating interferometry (XGI). It incorporated three major steps: denoising, the NSCT-SCM fusion algorithm, and enhancement. This strategy fulfilled the unique requirements of XGI: that the low-frequency coefficient should derive primarily from the AC channel in order to achieve final fusion results similar to traditional computed tomography (CT), and that the high-frequency coefficient should be selected in a way that preserves the details and features in the differential phase contrast and dark-field contrast channels.

Chuan Dai et al. [5] proposed a multi-layer perceptron (MLP)-based self-supervised learning framework with a contrastive learning loss function (ConMLP). ConMLP does not require a massive computational setup and can effectively reduce the consumption of computational resources. Compared with supervised learning frameworks, ConMLP is friendly to the huge amount of unlabeled training data. In addition, it has low requirements for system configuration and is more conducive to being embedded in real-world applications.

Ali Barzegar Khanghah et al. [6] presented a novel state-of-the-art 3D inflated CNN model to detect the correct and incorrect movement exercises performed by patients and provide them with feedback to refine their movements. The proposed model could achieve 90.57% accuracy and a 71.78% F1-Score for 10-fold validation. The proposed model can be readily used for assessing any exercise with a high accuracy at home, which can reduce potential costs, time, and the risk of infectious-disease transmission. Additionally, they expanded this platform for a better understanding of the rehab exercises by extracting features such as the inter-relationships of different parts of the body, such as muscles.

Panayiotis Antoniou et al. [7] investigated three different methods, along with different types of filtering techniques, for estimating the heart rate of individuals, using solely a smartphone camera to record a short video of the index finger attached directly to the camera. A series of measurements were performed on healthy human subjects, producing reliable data that compared favorably to benchmark data obtained by commercial and medically approved oximeters. Furthermore, the effect of the video recording duration on the accuracy of the results was investigated.

Jie Huang and Daqing Huang [8] designed and implemented a wearable body-temperature monitoring device, which was constructed by a graphene-enhanced polydimethylsiloxane patch and a temperature-measurement chip. The body-temperature patch adopts a completely flexible solution in combination with a near-field communication component, which provides the advantages of being passive wireless, flexible, and comfortable to wear. The whole device can be bent and stretched in conformal contact with skin. In order to improve the temperature-conduction ability of the polydimethylsiloxane patch and make the patch data more accurate, we adopted graphene nanoplates to significantly improve its thermal conductivity by 23.8%. This device can be utilized in multiple human-body-temperature-measurement scenarios and complex public-health situations.

Andrew Chen et al. [9] utilized a non-contact terahertz (THz) scanner designed for imaging spherical targets to discriminate between ex vivo corneal samples with intact and damaged endothelial layers. To create varying grades of corneal edema, the intraocular pressures of the whole porcine eye globe samples (*n* = 19) were increased to either 25, 35 or 45 mmHg for 4 h before returning to normal pressure levels at 15 mmHg for the remaining 4 h. Changes in tissue hydration were assessed by differences in spectral slopes between 0.4 and 0.8 THz. They demonstrated that THz can noninvasively assess the corneal endothelium and provide valuable complimentary information for the study and diagnosis of corneal diseases that perturb the hydration of the tissue.

Pengsu Mao [10] divided the health information that can be obtained from skin, using the sensor aspect’s input energy forms, into five categories: thermoelectrical signals, neural electrical signals, photoelectrical signals, electrochemical signals, and mechanical-pressure signals. They then summarized the current skin-wearable health-monitoring devices and provided outlooks on their future development. Skin-wearable sensors offer the opportunity to read health information non-invasively through human skin. Soft skin-electronic contacts reduce impedance and improve the signal-to-noise ratio. Tests of the reviewed sensors in this research have demonstrated the feasibility of their applications. The combination of technology, such as stretchable solar cells, triboelectric mechanisms, and bioelectrocatalytic reactions, with skin-wearable sensors could potentially make products more valuable.

Sandra Silva et al. [11] aimed to summarize the nonlinear measures used in the analysis of kinetic and kinematic data of human movement in healthy adolescents who were assessed in real-life environments. After the nonlinear measures were identified, the objective was to summarize the methodological considerations, namely the tasks under study, measurement instruments, and outcomes considered. The nonlinear measures identified included entropy (*n* = 8), fractal analysis (*n* = 3), recurrence quantification (*n* = 2), and the Lyapunov exponent (*n* = 2). This review demonstrated that, in adolescents assessed in a real context, entropy measures are preferred when studying the complexity of human movement, especially when examining multiscale entropy.

We would like to express our profound appreciation for the authors and reviewers who made this Special Issue possible. Our reviewers and authors have dedicated tremendous effort into the preparation of this Special Issue, in order to allow it come to life and flourish.
